# Plasma proteome of growing tumors

**DOI:** 10.1038/s41598-023-38079-9

**Published:** 2023-07-27

**Authors:** Shashi Gupta, Matthew J. Westacott, Deborah G. Ayers, Sophie J. Weiss, Penn Whitley, Christopher Mueller, Daniel C. Weaver, Daniel J. Schneider, Anis Karimpour-Fard, Lawrence E. Hunter, Daniel W. Drolet, Nebojsa Janjic

**Affiliations:** 1 SomaLogic, Inc., 2945 Wilderness Place, Boulder, CO 80301 USA; 2 Boulder BioConsulting, Inc., 325 S 68th St., Boulder, CO 80303 USA; 3 University of Colorado School of Medicine, Mailstop 8303, Aurora, CO 80045 USA

**Keywords:** Cancer, Tumour biomarkers, Computational biology and bioinformatics, Proteome informatics

## Abstract

Early detection of cancer is vital for the best chance of successful treatment, but half of all cancers are diagnosed at an advanced stage. A simple and reliable blood screening test applied routinely would therefore address a major unmet medical need. To gain insight into the value of protein biomarkers in early detection and stratification of cancer we determined the time course of changes in the plasma proteome of mice carrying transplanted human lung, breast, colon, or ovarian tumors. For protein measurements we used an aptamer-based assay which simultaneously measures ~ 5000 proteins. Along with tumor lineage-specific biomarkers, we also found 15 markers shared among all cancer types that included the energy metabolism enzymes glyceraldehyde-3-phosphate dehydrogenase, glucose-6-phophate isomerase and dihydrolipoyl dehydrogenase as well as several important biomarkers for maintaining protein, lipid, nucleotide, or carbohydrate balance such as tryptophanyl t-RNA synthetase and nucleoside diphosphate kinase. Using significantly altered proteins in the tumor bearing mice, we developed models to stratify tumor types and to estimate the minimum detectable tumor volume. Finally, we identified significantly enriched common and unique biological pathways among the eight tumor cell lines tested.

## Introduction

Detecting malignancies at an early stage remains a key unmet need in cancer diagnosis. For most cancers, early detection increases treatment options, minimizes the likelihood of resistance to chemotherapy, reduces the risk of metastases and, ultimately, improves long-term survival^1,2^. Nevertheless, in most cases, tumors are discovered at more advanced stages, either because of the onset of symptoms or as part of an unrelated procedure. Although screening tests for early detection are performed on five tumor types (mammography for breast cancer, colonoscopy or DNA test for colon cancer, pap smear for cervical cancer, PSA test for prostate cancer and chest X-ray or CT scan for lung cancer), their impact on cancer outcomes is still constrained by limited sensitivity, high false-positive rates (except in high-risk populations), and low compliance^3^.

Minimally invasive, repeatable means of detecting and characterizing tumors in systemic circulation through substances secreted or shed by growing tumors has long held considerable appeal but has also presented enormous challenges. A major difficulty is the assay sensitivity needed to detect the extremely low levels of biomarkers present in blood at the earliest stage of the disease. To date, such “liquid biopsies” have mainly focused on the identification of genetic material (mutations or epigenetic changes) unique to transformed cells, typically derived from circulating tumor cells, or cell-free DNA whose signal can be amplified by polymerase chain reaction or detected by next generation sequencing^4^.

The use of tumor-associated proteins for early detection has also been explored. In contrast to genetic testing, the signal obtained for a protein biomarker is more often a change in the circulating level of the unmutated protein (for example, PSA, CA 125). However, protein biomarkers cannot be amplified, and current detection methods are generally not as sensitive as tests that detect genetic material. A theoretical study which took into account the rates of protein synthesis and elimination concluded that it would be virtually impossible to detect solid tumors in humans with protein biomarkers in blood until the tumor has reached “the size of an olive”^5^. Aside from the obvious desire for better analytic sensitivity to enable earlier detection, knowing which proteins represent the earliest sentinel markers or tumor presence would be enormously useful. So what are the first tumor-associated proteins that can be detected in blood as the initial transformed cells establish residence in their tissues of origin, and can we use them to establish the presence and the identity of the tumors?

To address these questions, we monitored the time course of changes in the plasma proteome of individual mice carrying transplanted human tumors using the aptamer-based SomaScan assay that simultaneously measures ~ 5000 proteins^6,7^. The shortcomings of mouse models as surrogates of tumor growth in humans are well appreciated, but for this purpose, they offer an opportunity to follow changes in the proteome before implantation of tumor cells, as well as at multiple time points after tumor implantation. It is this longitudinal assessment in individual animals that allows correlation with tumor burden and differentiates our study from similar proteomic studies of tumor xenografts^8–12^.

Since lung cancer remains the leading cause of mortality from cancer^13^, we focused initially on two human non-small cell lung cancer (NSCLC) cell lines. We then expanded our investigation to include breast, colon, and ovarian cancer cell lines. The longitudinal emergence of a pattern of proteins identified in plasma of these tumor-bearing mice allowed us to identify biomarkers unique for each tumor type as well as shared biomarkers that can detect the presence and predict the volume of any of these tumors.

## Results

### Proteomic analysis of NSCLC tumor-bearing mice

**Figure 1:**
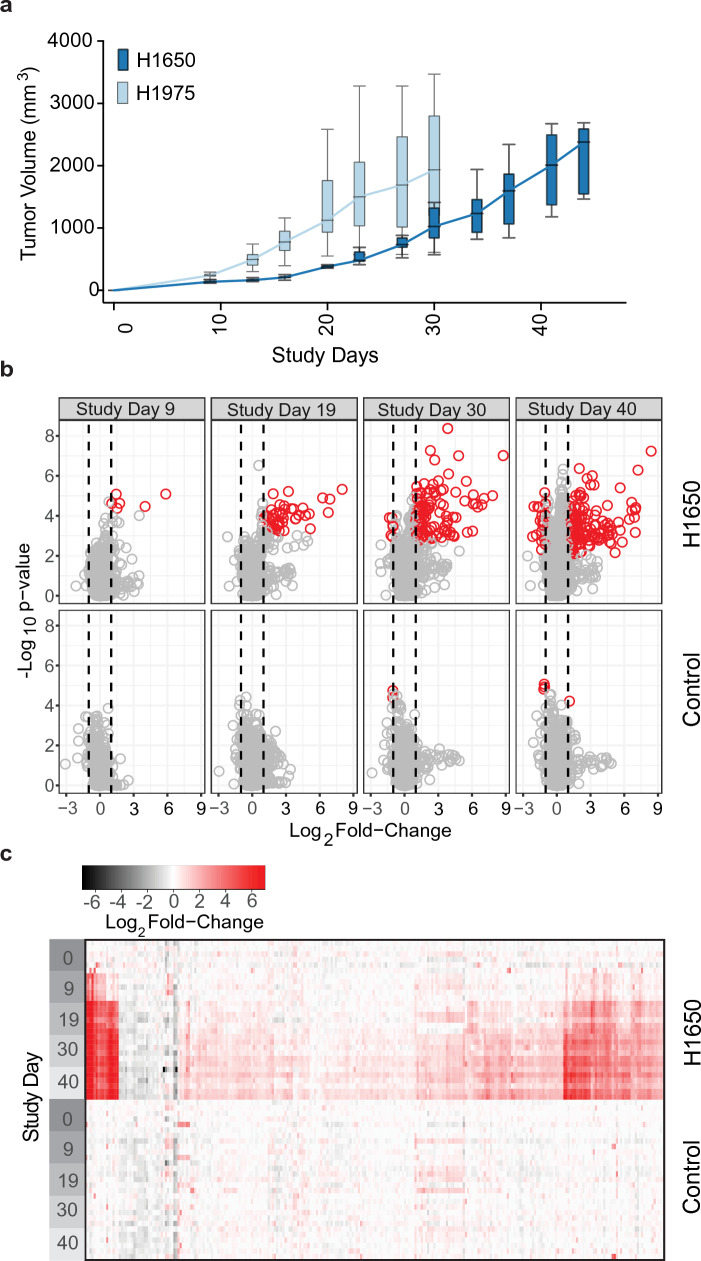
Impact of NSCLC tumor xenograft on the circulating proteome. (**a**) Tumor volume versus time for mice implanted subcutaneously with either H1650 or H1975 tumor cells (median, IQR with error bars representing 1.5 × IQR, n = 6/group). (**b**) Volcano plots showing the Log_10_ p-value versus median Log_2_ fold-change of 4584 individual analytes on different Study Days (9, 19, 30 and 40) relative to Study Day 0, for H1650 implanted mice (top panels) or for non-implanted (control) animals (bottom panels). Circles indicate individual analytes and vertical lines indicate a Log_2_ fold-change of |1|. Significant analytes (fdr corrected p-value ≤ 0.05 with median Log_2_ fold-change ≥|1|) are indicated by red circles. Volcano plots for H1975 tumors are shown in Supplementary Fig. S1. (**c**) Heatmap representing the fold changes of statistically significant analytes (as defined above). Individual animal Log_2_ fold-changes were calculated relative to the median values on Study Day 0 of control mice. For each Study Day indicated there are multiple rows, each representing an individual animal while each column represents a different protein analyte. Top panels show H1650 implanted mice and bottom panels show un-implanted (control) mice. Heatmap for H1975 tumors is shown in Supplementary Fig. S1.

**Figure 2:**
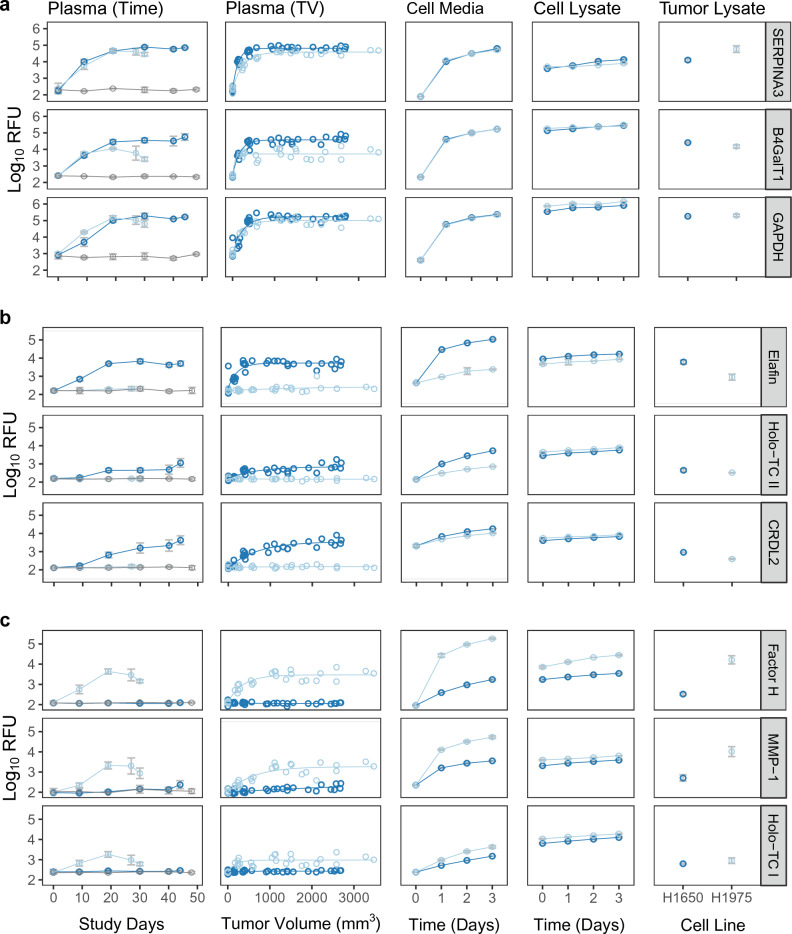
Circulating tumor volume prediction markers in H1650 and H1975 xenograft models. Signal in relative fluorescent units (RFU) versus Study Day [Plasma (Time)] and signal verses tumor volume [Plasma (TV)] are shown for a representative set of tumor volume prediction markers. Signals for the same markers from cell conditioned media versus time (Cell Media), cell lysate versus time, and end of study tumor lysate are also shown. Error bars indicate mean ± s.d., circles indicate individual measurements. Color scheme is same as in Fig. 1a. All markers showed statistically significant changes in signal with time or tumor volume using a repeated measured ANOVA (fdr corrected p-value ≤ 0.05). (**a**) Representative prediction markers shared between H1650 and H1975 models. (**b**) Representative markers specific to H1650. (**c**) Representative markers specific to H1975.

Erlotinib-sensitive (H1650) or erlotinib-resistant (H1975) NSCLC cells were implanted subcutaneously on study day 0 into the hind flank of female NCr mice and tumors were monitored until they reached a maximal size of 2 g (Fig. 1a). We analyzed protein levels in plasma samples collected from 5–6 individual animals on study days 0 (prior to implantation), 9, 19, 30, 40 and 44 for H1650 tumors and study days 0, 9, 19, 27 and 30 for H1975 tumors. Sampling time differences reflect an adjustment for differences in tumor growth rate. We identified 473 proteins in the slower-growing H1650 tumors and 1,345 proteins in the faster-growing H1975 tumors (Supplementary Table S1) that were statistically significant (fdr corrected p-value ≤ 0.05). Of these, 248 in H1650 and 159 in H1975 exhibited a fold-change from baseline ≥|2| (Fig. 1b, c; Supplementary Table S2). The distribution of changes was markedly skewed toward increased levels, as expected in mice with metabolically active tumors (Fig. 1b, c for H1650; Supplementary Fig. S1 for H1975). In comparison, 37 proteins changed significantly with time in control animals with no tumor cells implanted (Supplementary Table S3). After removing those proteins with significant change in the control group, we identified 98 statistically significant proteins in common between the two NSCLC xenograft models (Supplementary Table S4 and Fig. 2a). We also identified proteins with significantly increased expression unique to either the H1650 (150 proteins) or to the H1975 tumors (62 proteins) (Supplementary Table S5; Fig. 2b, c). To establish whether these biomarkers are expressed by H1650 and H1975 cells in culture, we measured the proteome changes of cell-conditioned media and cell lysates over several days (Fig. 2; Supplementary Tables S6 and S7). A substantial fraction of the significantly changed common plasma proteins could also be detected above background levels in either supernatants or cell lysates (98 and 99 percent in lysates; 63 and 73 percent in media of H1650 and H1975 lines, respectively), indicating that these biomarkers could be produced by the implanted tumors.

### Tumor detection from protein signatures of NSCLC tumors

**Figure 3:**
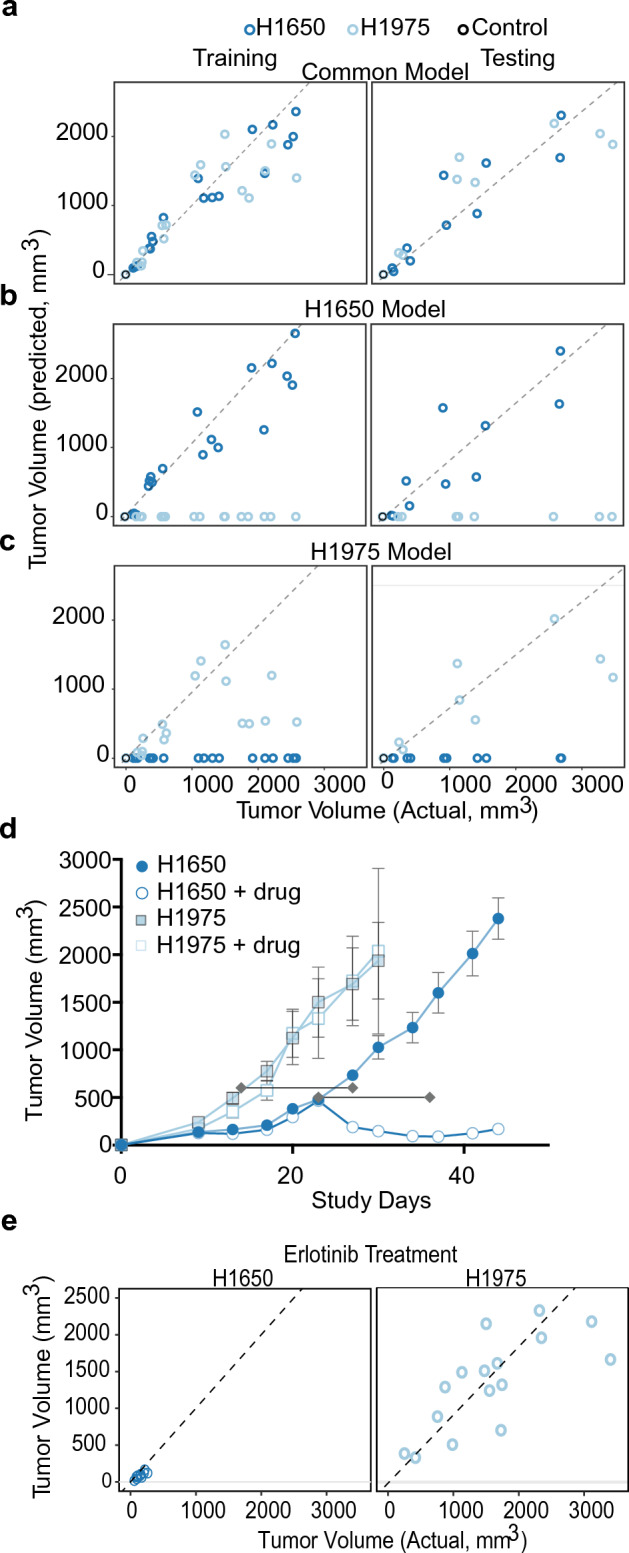
Tumor volume prediction in H1650 and H1975 tumor xenografts. (**a**) Actual versus predicted tumor volume trained using 19 common plasma markers between lung cell lines for the training set (n = 4 mice in each group) and testing set (n = 2 mice in each group) Dashed line indicates identity. Color scheme is the same as in Fig. 1A. Same analysis was used for data shown in panels (**b**) and (**c**). (**b**) Actual versus predicted tumor volume trained using 28 markers for H1650. (**c**) Actual versus predicted tumor volume trained using 3 markers for H1975. (**d**) Tumor volume versus time for mice implanted subcutaneously with either H1650 or H1975 tumor cells with or without erlotinib treatment (median, IQR with error bars representing 1.5 × IQR, n = 6/group). Horizontal lines indicate erlotinib treatment window. (**e**) Plot of actual versus predicted tumor volume with erlotinib treatment in both H1650 and H1975, using 19 common plasma markers. Dashed line indicates identity.

We used machine learning to build a model to differentiate tumor-bearing from non-tumor-bearing mice and to predict tumor volume. Using the common set of 98 statistically significant proteins shared between the H1650 and H1975 models (Supplementary Table S4) we trained an elastic net linear regression model, regressing on the tumor volume. This led to the selection of a 19-analyte model (common lung model) for tumor volume prediction (Supplementary Table S8). A training/testing split of 65/34 samples was used to train the model and evaluate model performance. There was strong correlation between actual versus predicted tumor volume with an explained variance (R^2^) for the training and testing sets of 0.90 and 0.87, respectively (Supplementary Table S9 and Fig. 3a). A degree of homoskedasticity in prediction (that is, increased scatter) is observed with larger tumor volumes which is expected when saturation of analyte signals occurs resulting in less discriminatory information at larger tumor volumes (Fig. 2).

The ability to differentiate tumor-bearing from non-tumor-bearing mice was evaluated by scanning the empirically predicted tumor volume on the holdout test set to define sensitivity/specificity metrics. Non-tumor bearing samples were derived from all time points of the control group and from study day 0 samples of the other groups (n = 16). Tumor volume prediction of non-tumor-bearing mice ideally should be centered at 0 mm^3^; however, the variability around the prediction for these mice may infer a lower limit for the minimum detectable tumor volume (MDTV) required for classification. For the common lung model, predicted tumor volumes < 0.75 mm^3^, an empirically predicted value of one of the samples, would classify the mouse as a non-tumor-bearing animal. If an animal was predicted to have a tumor size > 0.75 mm^3^ we would classify it as tumor-bearing mouse with a 94% specificity and 100% sensitivity (Supplementary Table S9).

To determine if tumor-prediction models are better using cell line-specific protein biomarkers, we repeated the analysis with biomarkers specific to either H1650- (150 proteins) or H1975-derived (62 proteins) tumors (Supplementary Table S5; Fig. 3b, c). To develop the H1650 model we used the same data set, elastic net method and training/testing split as above with the addition of manually imputing the H1975 tumor volumes to zero. This resulted in the selection of 28 analytes for H1650-derived tumor volume predictions (Supplementary Table S10). A similar method was used to select 3 analytes for H1975-derived tumor volume predictions (Supplementary Table S10). The performance of the cell line specific models was similar to the common-lung model with a training(testing) R^2^ of 0.94(0.86) and 0.62(0.81) for the H1650 and H1975 models, respectively (Supplementary Table S9). If an animal was predicted to have a tumor size > 0.22 mm^3^ and > 0.36 mm^3^ for H1650 and H1975 models, respectively, we would classify it as a tumor-bearing mouse (with a 96% specificity and 100% sensitivity).

We also assessed the ability of the common lung model to predict tumor volumes after erlotinib treatment for both erlotinib resistant H1975-derived tumors and for erlotinib sensitive H1650-derived tumors. Separate groups of mice bearing these tumors were administered a 100 mg/kg oral dose of erlotinib once daily for 14 consecutive days (n = 6/group). Treatment with erlotinib was initiated when tumors were approximately 500 mm^3^. As expected, tumor volumes decreased after drug treatment for H1650-derived tumors but not for H1975-derived tumors (Fig. 3d). The common lung tumor volume prediction model continued to perform well, accurately predicting volumes after erlotinib treatment for both tumor types (Fig. 3e). Compared to vehicle alone, signal changes were attenuated for many markers in the model for the erlotinib sensitive tumor (SERPINA3, β1,4-galactosyltransferase 1(B4GalT1), cystatin M, dihydrolipoamide dehydrogenase, mitochondrial (DLD), fibronectin fragment 3 (FN1.3), interleukin-15 receptor subunit alpha (IL-15 Ra), interleukin-8 (IL-8), kazal-type serine protease inhibitor domain-containing protein 1 (KAZD1), nucleoside-diphosphate kinase B (NDPK), plexin domain-containing protein 1 (PXDC1), protein S100-A6 (S100A6), and tenascin), but not for the resistant tumor (Supplementary Fig. S2).

### Tumor detection from plasma protein signatures of colon, breast, and ovarian tumors

**Figure 4:**
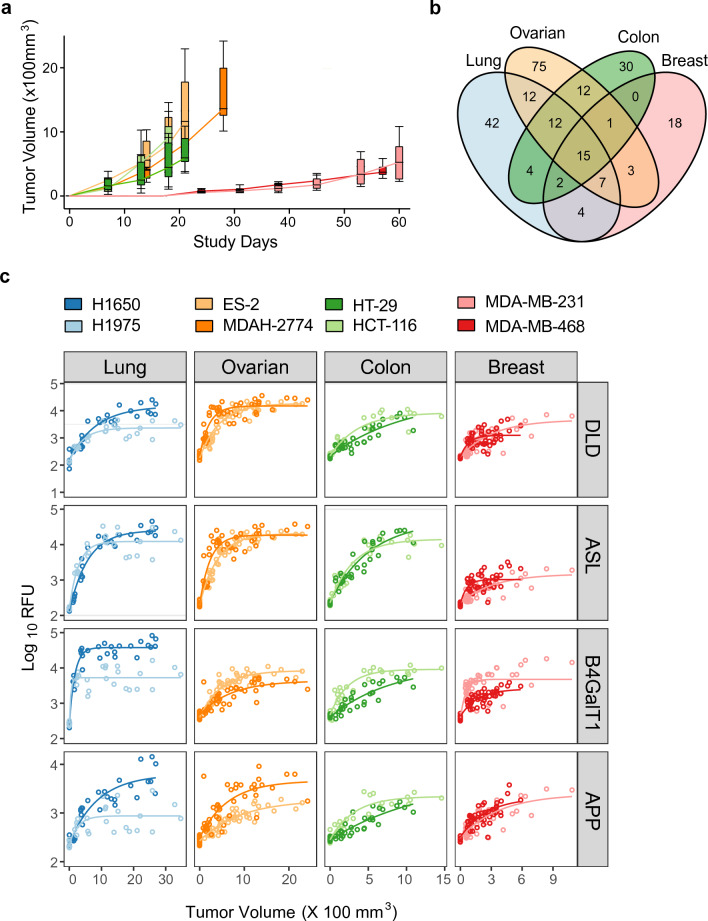
Impact of multiple tumor xenografts on the circulating plasma proteome. (**a**) Tumor volume versus time for mice implanted subcutaneously with either MDA-MB-231 or MDA-MB-468 (breast); ES-2 or MDAH-2774 (ovarian) and HCT-116 or HT-29 (colon) tumor cells (median, IQR with error bars representing 1.5 × IQR, n = 8, 12, 8 per cell line for breast, ovarian, and colon, respectively). (**b**) Venn diagram showing the numbers of statistically significant analytes (fdr corrected p-value ≤ 0.05 and a median Log_2_ fold-change ≥|1|) across all 8 human tumor xenograft models. (**c**) Plasma signal versus tumor volume for a set of the 4 common markers of tumor volume across tested cell lines. Circles indicate individual SomaScan assay measurements and lines indicate exponential fitting. The plots for the other 11 biomarkers are shown in Supplementary Fig. S4.

To identify proteomic changes in other cancer types, we performed additional xenograft studies using pairs of human breast (MDA-MB-231 and MDA-MB-468), ovarian (ES-2 and MDAH-2774), and colon (HCT-116 and HT-29) cancer cell lines (Fig. 4a). In all cases, we observed numerous significant changes in protein expression (fdr corrected p-value ≤ 0.05 with a fold-change of ≥|2|) from study day 0: 334 in ES-2 and, 427 in MDAH-2774 (ovarian cancer), 266 in HCT-116 and, 250 in HT-29 (colon cancer), 267 in MD-MBA-231 and 174 in MD-MBA-468 (breast cancer), excluding proteins that also changed significantly over time in control mice (Supplementary Table S11 for timepoints, Supplementary Tables S12–S14 with data for all analytes; Supplementary Tables S15–S17, ranked by fold-change). As with NSCLC tumors, we observed both unique and shared biomarkers for cell lines of the same tumor type. Some of the shared analytes were also present in other cancer types (Fig. 4b, c, Supplementary Tables S18–S21); others were unique for each cancer type (Supplementary Tables S22–S25). Using biomarkers shared among both cell lines of each tumor type led to prediction models of tumor presence for tumor volumes of > 0.03 mm^3^ for ovarian, 3.4 mm^3^ for breast and 15 mm^3^ for colon tumors with a 94% specificity and 100% sensitivity, and good concordance of actual versus predicted tumor volume in the training and testing datasets (Supplementary Tables S18–S20, S26, Supplementary Fig. S3).

### Tumor detection using plasma biomarkers shared among all tumor types

**Figure 5:**
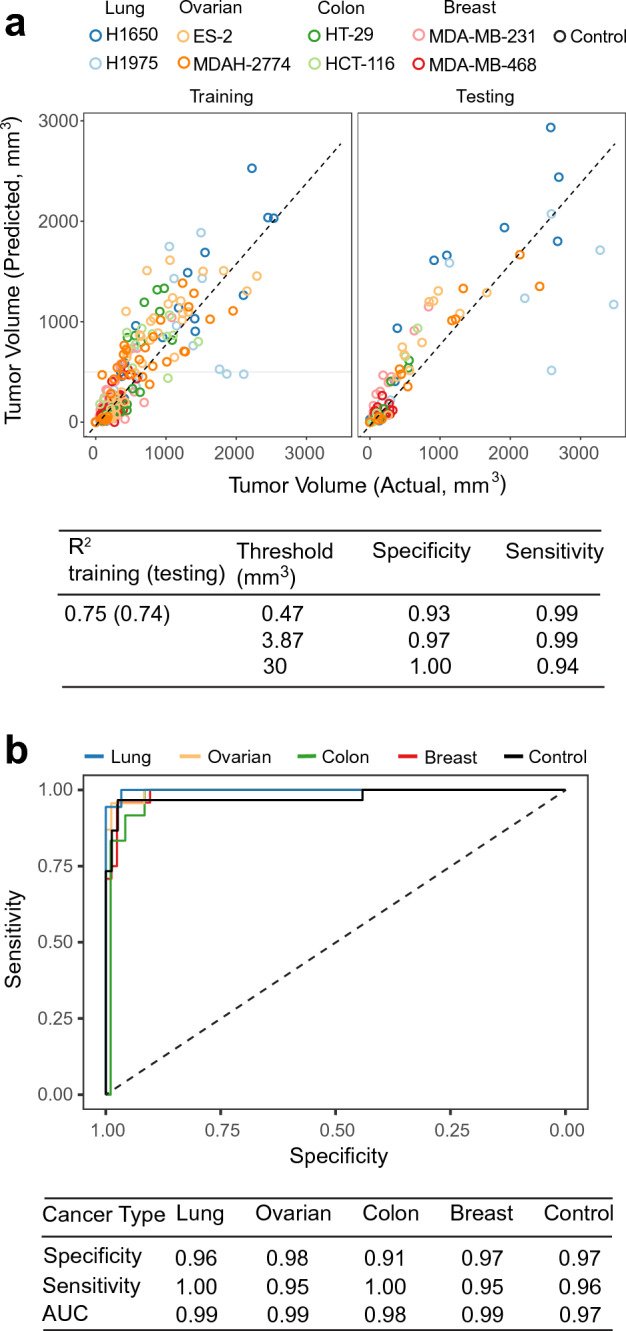
Tumor volume prediction and classification across cancer types. (**a**) Concordance of actual versus predicted tumor volume trained using 15 common analytes (Fig. 4b) obtained with linear regression with elastic net regularization. Result shown is the 27% hold-out test set. Dashed line indicates identity. (**b**) Receiver operating characteristic curves for tumor classification trained using 80 differentiating protein markers with elastic net regularization. Result shown is the 27% hold-out test set.

We identified 15 plasma biomarkers shared among all four tumor types and all eight cell lines (Supplementary Table S27, Fig. 4b, c and Supplementary Table S6). Using these biomarkers, we applied an elastic net linear regression model as described above to predict tumor volume using a training/testing split of 285/107 samples. There was good concordance of actual versus predicted tumor volume in the training and testing datasets of this pan cancer prediction model (Fig. 5a). If an animal was predicted to have a tumor volume > 3.9 mm^3^ it would be categorized as tumor bearing with 99% sensitivity and 97% specificity, a performance similar to that obtained with the tumor type specific models (Supplementary Table S28, Fig. 5a).

**Figure 6:**
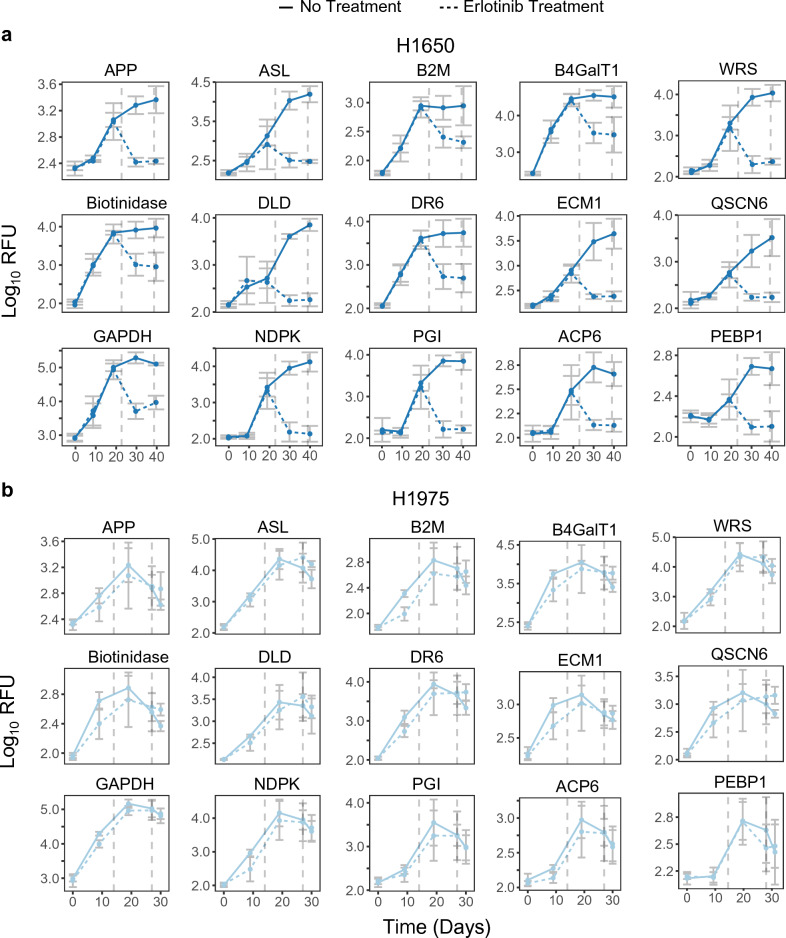
Impact of erlotinib treatment on plasma signal of 15 common markers. Signal in relative fluorescent units (RFU) versus Study Day for H1650 (**a**) and H1975 (**b**) animals and animals treated with erlotinib. Dashed vertical lines indicate start/stop of erlotinib treatment. Error bars indicate median + /− standard deviation.

This pan-cancer model also demonstrated the ability to predict tumor response to treatment. When animals bearing the erlotinib-sensitive H1650-derived lung tumor were treated with erlotinib, signals of all 15 of these biomarkers declined relative to the signals from animals bearing the same tumor but administered vehicle alone (Fig. 6). In contrast, there were no prominent differences in signal readout for these 15 biomarkers between the erlotinib treated and vehicle control groups for animals bearing the erlotinib-resistant H1975-derived tumor.

### Differentiation of tumor types

Although the previously described methods give an accurate prediction of tumor volume, they do not differentiate between the type of tumor. Towards this end, we used the unique biomarkers from each cancer type (Supplementary Tables S22–S25) and a multinomial logistic regression model across all cancer types and controls (Fig. 5b, Supplementary Table S29). The resulting model was highly sensitive (≥ 0.95) for selecting the correct tumor tissue (origin) type with mixed performance in specificity depending on the tumor type. The overall accuracy of selecting the correct tumor and differentiating non-tumor was 91%.

### Individual analytes that emerge in plasma at the earliest stages of tumor growth

**Figure 7:**
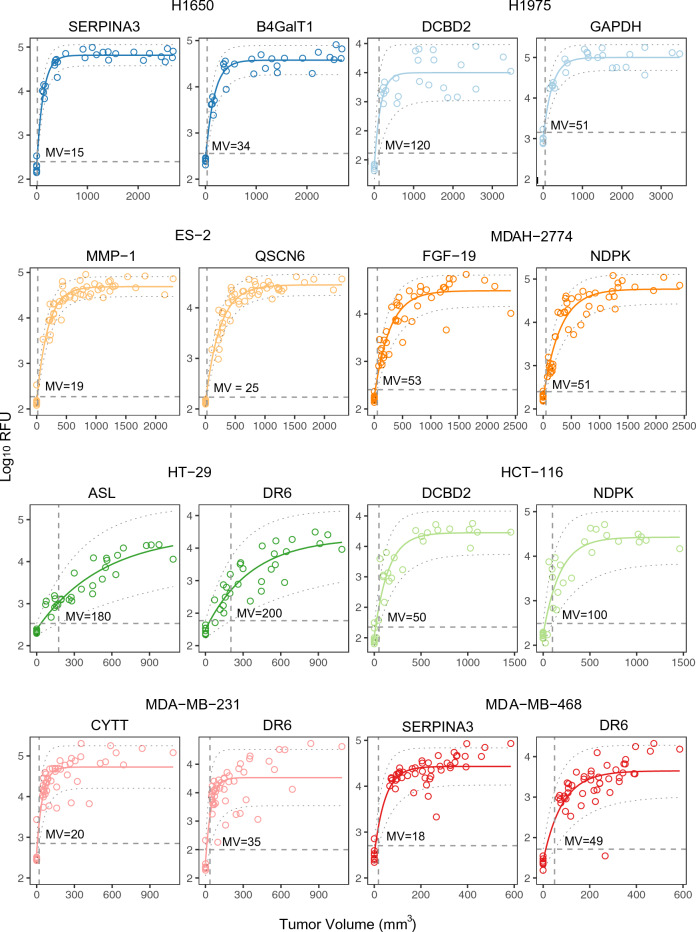
Minimum volume (MV) estimations across cancer types. Signal versus tumor volume for two representative analytes per cell line. Solid lines indicate non-linear fit and dashed lines indicate 95% CI. Vertical dashed line indicates estimated tumor volume detection threshold.

The changes in circulating proteins between the initial implantation day and the first day of observable tumor growth are massive, often spanning several orders of magnitude. Consequently, multivariate models can robustly differentiate tumor-bearing from non-tumor-bearing mice. Nevertheless, one could ask which individual proteins can be detected early, during the initial stages of tumor growth, for each tumor type, based on the rate of increase in plasma as a function of tumor volume. To answer this question, we selected proteins with the largest fold-changes that were statistically significant between pre-implantation and the first blood collection after implantation (Supplementary Table S11). Exponential growth curves were fit to each analyte signal with respect to tumor volume and estimates of the 95% confidence interval (CI) for the fit parameters were used to estimate a ‘lower limit of detection’ for tumor volume (Fig. 7 and Supplementary Table S30 for representative biomarkers and Supplementary Table S31 for the complete list). Several of the proteins whose level changes early for some tumor types (glyceraldehyde-3-phosphate dehydrogenase (GAPDH), B4GalT1, death receptor 6 (DR6, also known as tumor necrosis factor receptor superfamily member 21 (TNFRSF21), sulfhydryl oxidase 1 (QSCN6), arginosuccinate lyase (ASL), DLD, tryptophanyl-tRNA synthetase (WRS), biotinidase (BTD) and NDPK are among the 15 shared biomarkers that indicate the presence of any of the four tumor types studied. Generally, the MDTV estimates cover a range of tens to hundreds of cubic millimeters for different tumor types (Supplementary Table S31).

### Pathway analyses

**Figure 8:**
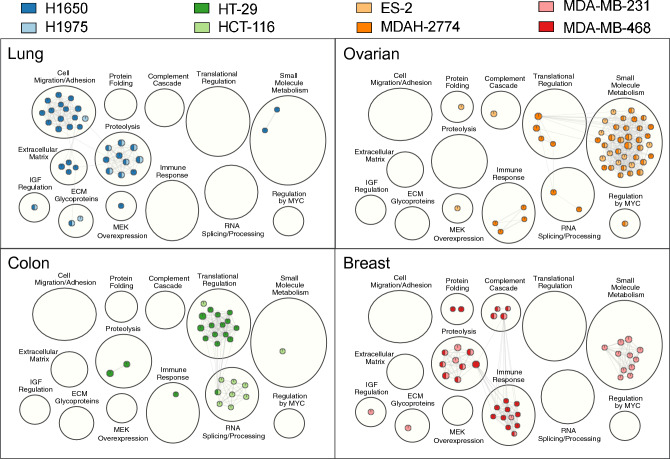
Pathway analysis delineates biological pathways in xenograft models stratified by tissue type. G:profiler pathway enrichment comparing study day 0 and the 60 timepoint in the eight cell line xenograft models. Cytoscape and Enrichment Map were used for clustering and visualization of the enrichment results. Nodes represent enriched gene sets, which are then clustered with related gene sets according to their gene content. Enrichment results were mapped as a network of gene sets (nodes). Node size is proportional to the total number of genes within each gene set. The proportion of shared genes between gene sets is represented as the thickness of the edge connecting nodes. The network map was manually curated by assigning functional categories to each cluster and by removing singleton gene sets. A complete list of enriched gene sets can be found in Supplementary Table S32, and each panel shows enriched gene sets for the 2 cell line xenografts for a given tissue type.

Pathway analysis was performed to identify significantly enriched GO biological processes and pathways across the eight xenograft models using significant analytes (fdr corrected p-value ≤ 0.05 and fold-change from baseline ≥|2|) (Supplementary Tables S2, S15–S17). The number and type of enriched pathways varied across xenografts (Fig. 8 and Supplementary Table S32). Ovarian xenograft cell line models had the greatest number of significantly enriched pathways (39 for MDAH-2774 and 35 for ES-2 cell lines) as well as the largest number of differentially expressed proteins (427 and 334 in MDAH-2774 and ES-2, respectively).

Enriched pathways for the ovarian xenografts could be grouped into several clusters, with small molecule metabolism related pathways being especially well represented followed by translational regulation, RNA processing and immune response pathways. Pathways detected in breast xenografts were also enriched for small molecule metabolism, as well as those involving proteolysis and the immune response. Proteolysis and cell migration/adhesion and extracellular matrix pathways were enriched in lung xenografts. Finally, colon xenografts had the fewest number of enriched pathways discovered and were mostly present in translational regulation and RNA processing clusters. The small molecule metabolism pathway was the only one to show significant enrichment in all four tissue types while the immune response and proteolysis pathways showed significant enrichment in 3 of the 4 tissue types. Additional functional pathways were detected in varying xenograft models. One of the striking features of each of the four tumor types is a clear pathway signature (Fig. 8), illustrating differences in the relative importance of distinct pathways for tumor growth.

## Discussion

There is a well-known direct correlation between tumor burden and metastatic risk^14^, tumor burden and resistance to chemotherapy^15^, and an inverse correlation between tumor burden and curability^16^. The importance of detecting tumors at early stages has therefore remained high, with much room for improvement over existing methods. Among other molecules, solid tumors (including tumor cells and associated stroma) produce proteins which end up in systemic circulation. For detection of such proteins in blood, a major difficulty is related to the need to detect a small volume of a malignancy remotely, after massive dilution of tumor-associated analytes in the total circulating volume of blood. In this study, we identify sentinel proteins in plasma for four different tumor types, each represented by two cell lines.

In both NSCLC cell lines, we were able to identify many highly statistically significant protein changes in plasma during the subcutaneous growth of transplanted tumors, with a strong bias toward increased protein levels. These changes included protein biomarkers shared between H1650 and H1975 tumors, as well as those unique to each tumor subtype. As tumors grow in the subcutaneous environment, changes in protein composition represent the cumulative contribution of proteins secreted by tumor cells, stromal cells, and to a lesser extent, proteins secreted from other organs, in response to the acute phase of tumor growth. To assess whether the tumor cells used for the xenograft studies could produce the proteins identified in plasma, we investigated the proteome that these cells produce in cell culture (both secreted proteins in the cell culture supernatant, as well as intracellular proteins from cell lysates). Most of the statistically significant common analytes identified in plasma of animals bearing H1650- or H1975-derived tumors were produced by these cells in culture. Nevertheless, it remains to be established whether the origin of these proteins are the tumor cells themselves or the host response to the tumors.

Predicting the presence of a tumor based on simultaneous measurement of multiple plasma proteins is possible using either common or unique biomarkers. Using the common biomarkers in a 19-analyte lung model, we could distinguish human lung tumor-bearing from non-tumor-bearing mice for tumors > 0.75 mm^3^. This model was also able to predict tumor volumes for both tumor cell lines before and after erlotinib treatment suggesting the possibility of developing more generalized clinically useful predictions of tumor burden from a simple blood test. Using biomarkers unique to each of the tumors resulted in prediction of tumors at a somewhat earlier, but overall comparable stage (> 0.22 and > 0.36 mm^3^, respectively).

How do these estimates compare to human tumor sizes? Accounting for the differences in blood volume between a mouse and a human, we estimate that the common lung model could detect spherical lung tumors around 1.7–2.0 cm in diameter (or about the size of an olive) while the cell-type specific models would do a bit better (1.1–1.3 cm). We get similar estimates when we use tumor weight to body weight ratios: 1.5–1.8 cm in diameter for common model and 0.9–1.2 cm for cell-type-specific models. These estimates are roughly equivalent to the minimum median detection estimates for circulating tumor DNA (ctDNA) of 2.0–2.3 cm^17^. Both estimates are better than the 3.5 cm current median detection size for lung tumors from the 2005 to 2015 SEER database as discussed in Avanzini et al.^17^.

We then extended these analyses to subcutaneous growth of cell lines representing ovarian, colon and breast tumors. As with NSCLC tumors, massive changes in the plasma proteome were detected with both shared and unique proteins readily identifiable in all the pairs of cell lines. Models analogous to those described for NSCLC tumors allowed the identification of tumors in the very small size range for ovarian tumors (> 0.03 mm^3^), and larger tumors for breast (> 3.4 mm^3^) and colon (> 15 mm^3^). Using the same adjustment for blood volume differences, this is equivalent to 0.6, 2.8 and 4.6 cm in diameter human tumors—a substantial range from very small to medium size tumors.

Using biomarkers shared among all tumor types we developed a pan cancer prediction model that can detect the presence of any of these tumors at volumes of  > 3.9 mm^3^. Many of these non-tumor differentiating biomarkers were among the group with the largest early changes. Tumor type can subsequently be established using proteins unique to each tumor. These observations demonstrate that a general-to-specific tumor type identification approach is feasible, without a substantial compromise in the ability to detect tumors at early stages.

For tumor growth and invasion, cancer cells evolve toward acquiring several capabilities: maintenance of proliferative state, avoidance of apoptosis, increase in angiogenesis, activation of invasion/metastasis, continuous replication, and avoidance of tumor suppressors^18,19^. These malignant cells reprogram their metabolic pathways to sustain their need for energy and building blocks for tumor growth. Indeed, of the 15 proteins in the pan cancer model (Supplementary Table S27), three (GAPDH, phosphoglucose isomerase (PGI), and DLD) are important for energy metabolism and seven (ASL, WRS, NDPK, QSCN6, B4GalT1, lysophosphatidic acid phosphatase type 6 (ACP6), and BTD) are important for maintaining protein, lipid, nucleotide, or carbohydrate balance (Supplementary Table S33). However, many of these proteins (GAPDH, PGI, DLD, ASL, WRS, and NDPK) are multifunctional, or so called moonlighting proteins, involved in a growing list biological pathways including tumorigenic pathways^20–29^ and several shown to have altered expression levels in some human cancers^30–38^. An example of note is WRS which catalyzes the loading of tryptophan onto its cognate tRNA. Since tryptophan is the least abundant of the proteinogenic amino acids, it may play a rate-limiting role during protein synthesis, so the increase in WRS in tumor cells could globally accelerate protein synthesis^39^. Depletion of tryptophan in tumors results in post-transcriptional tryptophan-to-phenylalanine substitution in proteins through codon reassignment^40^. Interferon gamma can upregulate the synthesis and secretion of WRS from endothelial cells, fibroblasts, and macrophages where it interacts with TLR2 and/or TLR4 on macrophages stimulating the innate immune response^29,41^. WRS also has roles in the extracellular domain: following cleavage by plasmin and/or elastases, extracellular WRS acts on endothelial cells to inhibit angiogenesis via interaction with VE-cadherin^29^.

Significantly enriched pathways in the xenograft models varied by tissue type, and to a lesser extent by the specific cell line used to construct the xenograft (Fig. 8). Enriched pathways fell into one of several functional clusters, including small molecule metabolism, translational regulation, RNA processing, immune response, proteolysis, cell migration or adhesion and extracellular matrix. Additional signaling pathways were also detected. Although we found some functional clusters specific to a given tissue, for example extracellular matrix and cell migration/adhesion pathways were lung specific, most pathway groups were found in more than one tissue type. These results also suggest a concerted biological response shared across different xenograft systems.

Our study has several limitations. Since this was a longitudinal mouse study, plasma volumes were small and insufficient for orthogonal validation of these biomarkers using the samples from this study. However, the aptamers used for measuring 188 of the 236 analytes that are significantly different with at least a twofold change in our study have had at least one previously performed orthogonal confirmation of their binding specificity (see Supplementary Table S21). For example, all 15 biomarkers that are shared among all cancer types in our study, are responsive to mutations in the genes that encode them (that is, all have exhibited cis pQTL associations). In addition, 13 of these 15 biomarkers have also been confirmed by mass spectrometry (enrichment of a cognate protein from a biological sample, either human plasma, serum, urine, or cell culture) as well as other orthogonal specificity confirmations (Supplementary Table S21). We also do not have information about the impact of post-translational modifications on the binding affinity of SOMAmer reagents used in the SomaScan assay.

This study identifies early sentinel proteins of tumor presence and estimates the MDTV that can also be stratified using protein biomarkers in blood. Since each tumor arises from clonal expansion of cells within an organ or tissue but with diverse paths to transformation, we observe both common and unique protein signatures, within as well as across tumor types. In this context, however, it is also important to note other limitations of this study: these models use established cell lines that are not grown in their native microenvironment which could have an impact on both the tumor cell expression pattern and on the expression pattern of the surrounding normal tissue. Perhaps even more importantly, the tumor cells used for implantation in this study are in a more advanced state of selection/evolution at the time they were collected from human patients than would be typically present in the earliest true cancerous cell population or in a precancerous cell population with changes indicating a likely trajectory to cancer^2^. At this time, we do not have appropriate matching human blood samples from early-stage malignancies for comparison to the mouse data reported in this study. Nonetheless, our study provides important insights into the use of protein biomarkers for early detection, progression, and stratification of cancer, including treatment based on the changing proteome in xenografts transplanted with patient-derived tumors.

## Methods

All methods are reported in accordance with the ARRIVE guidelines.

### Reagents

Media (McCoy’s 5a Modified, RPMI-1640, Leibovitz’s L-15, Dulbecco's Modified^39^ Eagle Medium, M-PER™ (# 78501), and T-PER™ (Cat# 78510) lysis buffers, GlutaMAX, Penicillin/Streptomycin, Fetal Bovine Serum (FBS), BCA Protein Assay Kit (Cat# 23235) and Halt™ (Protease/phosphatase Inhibitor Cat# 78429) were purchased from Thermo Scientific™. Captisol and erlotinib were purchased from CyDex Inc. (Cat# NC-04A-05009) and LC Laboratories (E-4007), respectively. The polyanionic competitor, Z-Block, was synthesized in-house and is a single stranded modified DNA [5′- (AC-BnBn)_7_-AC-3′], where Bn indicates a 5-benzyl-substituted deoxyuridine residue. Assay dilution buffer is composed of 66.8 mM HEPES pH 7.5, 5 mM KCl, 10.3 mM MgCl_2_, 16.7 mM EGTA, 1.8% tween 20, 2.2 mM benzamidine and 33.4 µM Z-block. The assay buffer is composed of 40 mM HEPES pH 7.5, 5 mM KCl, 5 mM MgCl_2_, 1 mM EDTA and 0.05% TWEEN 20 (Sigma-Aldrich).

### Cell culture

Cell lines were obtained from the American Type Culture Collection (ATCC) and cultured as per ATCC recommendations (Supplementary Table S11): NCI-H1650 (CRL-5883), NCI-H1975 (CRL-5908), HT-29 (HTB38), HCT-116 (CCL-247), MDA-MB-468 (HTB-132), MDA-MB-231 (HTB-26) and ES-2 (CRL-1978). MDAH-2774 (CRL-10303) were purchased from AddexBio, San Diego, CA.

### Animal procedures

All animal procedures were approved by the Institutional Animal Care and Use Committee at Inotiv-Boulder, Inc. (Boulder, CO) and conducted in accordance with all state and federal guidelines. Female NCRNU mice (5–6 weeks old) were obtained from Envigo and acclimatized for seven days (18–26 °C, a relative humidity 30–70%;12-h light/dark cycle). Food and water were provided ad libitum. Animals were randomly assigned to groups by body weight and tumors were established by injecting 0.5–1 × 10^7^ cells per animal in a single subcutaneous site in the flank (5–12/group; Supplementary Table S11). Lung tumors were treated with erlotinib (100 mg/kg) or vehicle control (6% Captisol in water) administered orally once daily for 14 consecutive days beginning on study day 14 for H1975 tumors and on study day 23 for H1650 tumors. Dose volumes were calculated using the most recent body weight. Animals were monitored daily for morbidity/mortality and body weights and tumor volumes measured twice weekly. Volumes were calculated using the formula: length × (width)^2^/2. At end of study, euthanasia was performed according to the AVMA guidelines for the euthanasia of animals. Anesthesia was first induced by 3% isoflurane followed by exsanguination and pneumothorax.

### Plasma and tumor collection

Ethylenediaminetetraacetic acid (EDTA) blood samples were collected from orbital sinus bleeds from anesthetized mice. Plasma was obtained by centrifugation at 3600×*g* for 10 min at 4 °C then frozen (− 80 °C). On the last day (Supplementary Table S11) animals were sacrificed, tumors excised, weighed, and frozen in liquid nitrogen.

### H1650 and H1975 cell culture

Cells (1 × 10^5^ cells) (Supplementary Table S11) were plated in media containing 10% FBS and placed in a humidified 37 °C incubator (5% CO_2_). Media was aspirated daily and control media (growth media with 0.1% FBS and 0.1% DMSO) added to each well. After a further 24-h incubation, the cell-conditioned media was collected (Day 1) from 3 individual wells and each centrifuged for 5 min at 16,000×*g* (4 °C). Supernatants were stored at − 80 °C. Unconditioned media was used as the day 0 control. For cell lysates, media was aspirated, cells washed twice with ice-cold PBS. M-PER lysis buffer (250 µL) containing HALT was added for 2 min at 4 °C while gently rotating the sample. Cells were removed by scrapping and debris pelleted by centrifugation (5 min at 16,000×*g*, 4 °C. Supernatants were stored at − 80 °C. To remaining wells, growth media with 0.1% FBS and 0.1% DMSO was added, and the cycle repeated for the Day 2 and 3 collections.

### Tumor lysates

Frozen tumors (10–20 mg) were pulverized (BioSpec Products 59012N Tissue Pulverizer; # UX-36903-00) and thawed on ice. Twenty volumes of T-Per with HALT were added and samples homogenized (DMK Life Sciences Tissue Grinder; #K749540-0000) for 5 min (4 °C). Samples were centrifuged and supernatants stored as described above.

### Proteomic analyses

Plasma proteomic analyses were conducted using using a 3-dilution (5%, 2%, and 0.05%) SomaScan assay generally as described^6,42^. Briefly, 11 µL of each sample was mixed with 2.5 µL of 27 mM EDTA followed by the addition of 132 µL of sample diluent and 74.5 µL of assay buffer. The resulting mixture, containing 5% plasma, was added to the first assay plate (100 µL). The remainder was diluted serially to 2% and 0.5% plasma using assay buffer and 100 µL of each added to the second and third assay plates, respectively. Cell and tumor lysate samples were run in a single dilution assay at 20 µg/mL protein in assay buffer containing 1 × HALT and 0.5 µM Z-block using the same assay version but with Agilent slides with fewer anti-aptamer probes. Protein concentrations were determined by the micro-BCA method. Cell conditioned media samples were run in a single dilution assay after diluting fivefold into assay buffer containing 1 × HALT and 0.5 µM Z-block. The SOMAscan assay (v3) used in this study is based upon a mixture of slow off-rate modified aptamers (SOMAmer^®^ reagents) targeting about 5,000 human proteins. Each reagent contains a 5′-fluorophore (cyanine 3), a biotin, and a photocleavable linker. This mixture was immobilized on streptavidin-coated beads and incubated for 2 h at 28 °C with sample concentrations and buffers as described above such that each SOMAmer reagent was at ~ 0.5 nM. After washing with assay buffer, bound proteins were biotinylated and the SOMAmer reagents released from the beads using ultraviolet light. A kinetic challenge was applied by adding a polyanionic competitor to the mixture to enrich for specific protein:SOMAmer complexes which have slower off-rates than any non-specifically bound SOMAmer reagents. Those complexes that survived kinetic challenge and free proteins, were then captured on streptavidin beads. After further washing, protein bound SOMAmer reagents were eluted from the beads and quantified by hybridization to a DNA array (Agilent) containing an anti-SOMAmer DNA probe for each SOMAmer reagent in the original mixture. Target protein concentration in the sample is proportional to the fluorescent intensity of each feature of the array as measured via the cyanine 3 dye. All samples were randomized on the assay plates and assay operators were blinded to the study groups. Information on assay cross reactivity to mouse proteins is given in the Supplementary Note and Supplementary Fig. S5).

### Data pre-processing

Variation in hybridization efficiency was normalized using the median fluorescent ratio of a set of 12 independent hybridization control oligonucleotides added at fixed concentrations that span the dynamic range of the Agilent reader. Plate to plate variations were corrected using 5 mouse EDTA-plasma calibrator samples per plate. Calibration scale factor ratios were calculated from the median signal for each SOMAmer reagent from the mouse calibrators to a fixed reference and applied to all mouse samples on the plate. Variability in overall sample intensity was corrected on a sample-by-sample basis, and by dilution group, by calculating the median ratio of each analyte relative to the intra-plate median and using the median value of those ratios as a scale factor applied to the individual sample.

### Statistical analyes

Statistical analyses were carried out in R (3.5.2). Assay signals were Log_10_ transformed prior to inference testing. Analyst was not blinded to the group identities.

### Plasma data

For each cell line and analyte, repeated measures analysis of variance (RMNOVA) was used first to identify statistically significant changes with respect to study days using each animal as the repeated measure. p-values were adjusted for multiple comparisons using the Benjamini–Hochberg technique. Analytes with an adjusted p-value ≤ 0.05 and a median Log2-fold change ≥|1| relative to the signal on study day 0 were preliminarily selected for inclusion into models. For further pruning to prevent false positives, we evaluated Pearson’s correlation between tumor volume and analyte signal. Analytes were removed from inclusion if Pearson’s correlation coefficient was <|0.25|.

### Conditioned media and lysates

ANOVA was used to identify statistically significant changes of analyte signal with respect to study days for each lung cell line (3/line/time-point). p-values were adjusted for multiple comparisons as above and median Log_2_-fold change determined relative to day 0.

### Tumor lysates

Differential expression for each analyte between excised lung cancer tissues was evaluated by Student’s unpaired t-tests (n = 3/tumor). Analytes with fdr corrected p-value ≤ 0.05 and above a median Log_2_-fold change ≥|1| are reported.

### Tumor size and classification prediction models

Elastic net regression models (linear and logistic) were trained using ‘Caret’ (6.0.86)^43^ in R allowing the mixing parameters, and the regularization parameter, to grid-search over [0, 1]. Prior to training for tumor size prediction, the tumor volume was transformed with a cubic root and raw RFU values Log_10_ transformed, centered, and scaled. A 20% hold out set was used to evaluate model performance metrics. MDTV predictions were estimated by nonlinear modeling of the signal of an analyte with respect to tumor volume defined by:

$$RFU=A*\left(1-{e}^{-B*Volume}\right)+C.$$

Parameters A, B, and C were estimated through nonlinear least squares R package stats 3.6.3. MDTV was estimated by first establishing 95% CIs around the signal of non-tumor bearing mice for each cell line. RFU variance, with respect to tumor volume, was assumed to be constant and MDTV calculated by inverting the previous equation and determining the volume at which the lower 95% CI the absence of any tumor intersected the upper 95% CI.

### Pathway analyses

Analysis of enriched pathways was performed for the eight xenograft models. Differentially expressed proteins (fdr q ≤ 0.05 and Log_2_-fold-change >|2|), excluding analytes which changed in control mice, were used as input for the g:profiler pathway enrichment method using gprofiler2.v0.2.1 in R^44,45^. Annotated gene sets representing Gene Ontology biological processes, Hallmark pathways, C6 Oncogenic signature gene sets and Canonical Pathways (incl. Biocarta, KEGG, PID, Reactome and Wikipathways) were downloaded from the Molecular Signatures Database v7.5.1 for use as target pathways^46^. Due to the semi-targeted nature of the SomaScan protein quantification method a unique list of all proteins interrogated on the platform (n = 4132) were used as a statistical null background. An fdr threshold of q < 0.25 was used to select enriched gene sets for further analysis. The Cytoscape Enrichment Map plugin v3.3.4^47^ was used for clustering and visualization of significantly enriched gene sets detected for each xenograft model. A Jaccard plus overlap combined coefficient of 0.375 was used for defining Default cluster edges (default setting). Singleton gene sets were removed from visualization if a single cell line model contributed to enrichment and the gene set was not connected to a larger functional cluster.

## Supplementary Information

Supplementary Information 1.

Supplementary Information 2.

## Data Availability

All summary data are included in this manuscript (and its Supplementary Information files). All raw proteomics data will be made available from the corresponding author on reasonable request.

## References

[1] Vasan N, Baselga J, Hyman DM (2019). A view on drug resistance in cancer. Nature.

[2] Crosby D (2022). Early detection of cancer. Science.

[3] Shieh Y (2016). Population-based screening for cancer: Hope and hype. Nat. Rev. Clin. Oncol..

[4] Alix-Panabières C, Pantel K (2021). Liquid biopsy: From discovery to clinical application. Cancer Discov..

[5] Hori SS, Gambhir SS (2011). Mathematical model identifies blood biomarker-based early cancer detection strategies and limitations. Sci. Transl. Med..

[6] Gold L (2010). Aptamer-based multiplexed proteomic technology for biomarker discovery. PLoS ONE.

[7] Schneider, D. J. *et al.* In *RNA Therapeutics* (eds Giangrande, P.H., de Franciscis, V., & Rossi, J.J.) 171–260 (Academic Press, 2022).

[8] Tang HY (2012). A xenograft mouse model coupled with in-depth plasma proteome analysis facilitates identification of novel serum biomarkers for human ovarian cancer. J. Proteome Res..

[9] Taguchi A (2011). Lung cancer signatures in plasma based on proteome profiling of mouse tumor models. Cancer Cell.

[10] Tveitarås MK, Selheim F, Sortland K, Reed RK, Stuhr L (2019). Protein expression profiling of plasma and lungs at different stages of metastatic development in a human triple negative breast cancer xenograft model. PLoS ONE.

[11] Hood BL (2005). Quantitative analysis of the low molecular weight serum proteome using 18O stable isotope labeling in a lung tumor xenograft mouse model. J. Am. Soc. Mass Spectrom..

[12] Beer LA (2013). Identification of multiple novel protein biomarkers shed by human serous ovarian tumors into the blood of immunocompromised mice and verified in patient sera. PLoS ONE.

[13] Mattiuzzi C, Lippi G (2019). Current cancer epidemiology. J. Epidemiol. Glob. Health.

[14] Fisher B, Slack NH, Bross ID (1969). Cancer of the breast: Size of neoplasm and prognosis. Cancer.

[15] Goldie JH, Coldman AJ (1979). A mathematic model for relating the drug sensitivity of tumors to their spontaneous mutation rate. Cancer Treat. Rep..

[16] Goldie JH, Coldman AJ (1984). The genetic origin of drug resistance in neoplasms: Implications for systemic therapy. Cancer Res..

[17] Avanzini S (2020). A mathematical model of ctDNA shedding predicts tumor detection size. Sci. Adv..

[18] Hanahan D, Weinberg R (2011). Hallmarks of cancer: The next generation. Cell.

[19] Hanahan D, Weinberg RA (2000). The hallmarks of cancer. Cell.

[20] Adamo A (2020). Moonlighting proteins are important players in cancer immunology. Front. Immunol..

[21] Dayan A, Yeheskel A, Lamed R, Fleminger G, Ashur-Fabian O (2020). Dihydrolipoamide dehydrogenase moonlighting activity as a DNA chelating agent. Proteins.

[22] Singh N, Bhalla N (2020). Moonlighting proteins. Annu. Rev. Genet..

[23] Tarrado-Castellarnau M (2017). Glyceraldehyde-3-phosphate dehydrogenase is overexpressed in colorectal cancer onset. Transl. Med. Commun..

[24] Min KW, Lee SH, Baek SJ (2016). Moonlighting proteins in cancer. Cancer Lett..

[25] Lagana A, Goetz JGYN, Altschuler Y, Nabi IR (2005). pH-specific sequestration of phosphoglucose isomerase/autocrine motility factor by fibronectin and heparan sulphate. J. Cell Sci..

[26] Jeffery CJ (2011). Proteins with neomorphic moonlighting functions in disease. IUBMB Life.

[27] Sirover MA (2018). Pleiotropic effects of moonlighting glyceraldehyde-3-phosphate dehydrogenase (GAPDH) in cancer progression, invasiveness, and metastases. Cancer Metastasis Rev..

[28] Jeffery CJ (2020). Enzymes, pseudoenzymes, and moonlighting proteins: Diversity of function in protein superfamilies. FEBS J..

[29] Jobin PG (2019). Matrix metalloproteinases inactivate the proinflammatory functions of secreted moonlighting tryptophanyl-tRNA synthetase. J. Biol. Chem..

[30] Kang UB (2010). Differential profiling of breast cancer plasma proteome by isotope-coded affinity tagging method reveals biotinidase as a breast cancer biomarker. BMC Cancer.

[31] Zhou Z, Sun B, Nie A, Yu D, Bian M (2020). Roles of aminoacyl-tRNA synthetases in cancer. Front. Cell Dev. Biol..

[32] Lake DF, Faigel DO (2014). The emerging role of QSOX1 in cancer. Antioxid. Redox Signal.

[33] Xie H (2016). Increased B4GALT1 expression associates with adverse outcome in patients with non-metastatic clear cell renal cell carcinoma. Oncotarget.

[34] Huang HL (2013). Attenuation of argininosuccinate lyase inhibits cancer growth via cyclin A2 and nitric oxide. Mol. Cancer Ther..

[35] Thakur RK, Yadav VK, Kumar P, Chowdhury S (2011). Mechanisms of non-metastatic 2 (NME2)-mediated control of metastasis across tumor types. Naunyn Schmiedebergs Arch. Pharmacol..

[36] Chryplewicz A (2019). Mutant p53 regulates LPA signaling through lysophosphatidic acid phosphatase type 6. Sci. Rep..

[37] Zhu X (2005). Elevated beta1,4-galactosyltransferase I in highly metastatic human lung cancer cells. Identification of E1AF as important transcription activator. J. Biol. Chem..

[38] Di Giovanni S, Valentini G, Carducci P, Giallonardo P (1989). Beta-2-microglobulin is a reliable tumor marker in chronic lymphocytic leukemia. Acta Haematol..

[39] Adam I (2018). Upregulation of tryptophanyl-tRNA synthethase adapts human cancer cells to nutritional stress caused by tryptophan degradation. Oncoimmunology.

[40] Pataskar A (2022). Tryptophan depletion results in tryptophan-to-phenylalanine substitutants. Nature.

[41] Jin M (2019). Unique roles of tryptophanyl-tRNA synthetase in immune control and its therapeutic implications. Exp. Mol. Med..

[42] Williams SA (2019). Plasma protein patterns as comprehensive indicators of health. Nat. Med..

[43] Kuhn M (2008). Building predictive models in R using the caret package. J. Stat. Softw..

[44] Kolberg L, Raudvere U, Kuzmin I, Vilo J, Peterson H (2020). gprofiler2—An R package for gene list functional enrichment analysis and namespace conversion toolset g:Profiler. F1000Res.

[45] Raudvere U (2019). g:Profiler: A web server for functional enrichment analysis and conversions of gene lists (2019 update). Nucleic Acids Res..

[46] Liberzon A (2011). Molecular signatures database (MSigDB) 3.0. Bioinformatics.

[47] Merico D, Isserlin R, Stueker O, Emili A, Bader GD (2010). Enrichment map: A network-based method for gene-set enrichment visualization and interpretation. PLoS ONE.

