# Supplementary material for: Plasma proteome of growing tumors
Source: Sci Rep. 2023 Jul 27;13:12195. doi: 10.1038/s41598-023-38079-9 (PMC10374562; doi:10.1038/s41598-023-38079-9)
Supplement: Supplementary file 2 — Supplementary Information 2. [file 41598_2023_38079_MOESM2_ESM.docx]

**Plasma proteome of growing tumors**

Shashi Gupta^1,4^, Matthew J. Westacott^1,4^, Deborah G. Ayers^1^, Sophie J. Weiss^1^, Penn Whitley^2^, Chris Mueller^2^, Daniel Weaver^2^, Daniel J. Schneider^1^, Anis Karimpour-Fard^3^, Lawrence E. Hunter^3^, Daniel W. Drolet^1^, Nebojsa Janjic^1^*

^1^SomaLogic, Inc., 2945 Wilderness Place, Boulder, CO 80301, USA

^2^Boulder BioConsulting, Inc. 325 S 68th St. Boulder, CO 80303, USA

^3^University of Colorado School of Medicine, Mailstop 8303, Aurora, CO 80045, USA

^4^These authors contributed equally

*Corresponding author

Email: njanjic@somalogic.com

**SUPPLEMENTARY FIGURES**


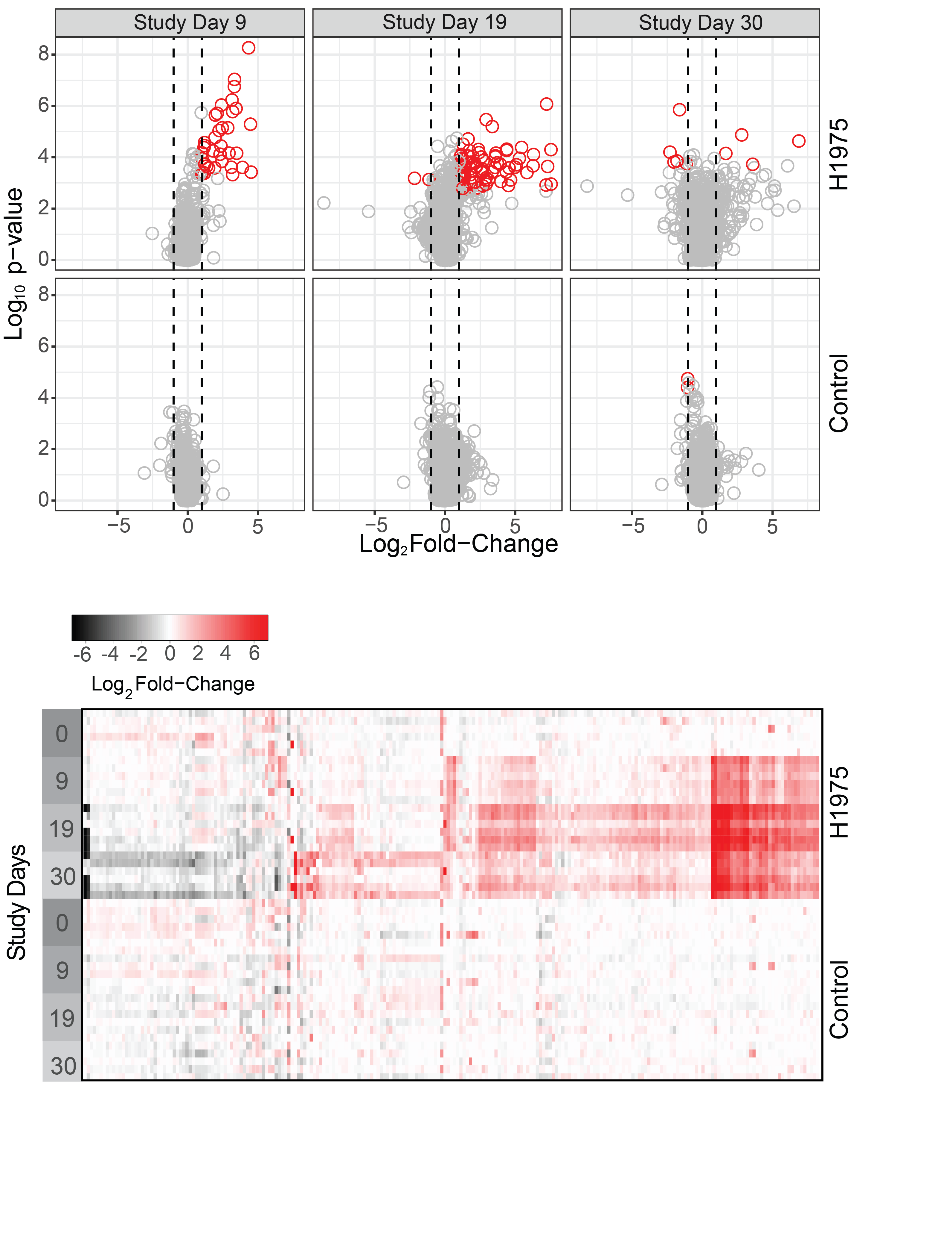


**Figure S1**. Impact of NSCLC tumor xenograft model on the circulating plasma proteome of mice. Volcano plots (top panels) showing the -Log_10_ p-value versus median Log_2_ fold-change in 4,584 individual analytes on different Study Days (9, 19 and 30) relative to Study Day 0, for H1975 implanted mice or for non-implanted (control) animals. Circles indicate individual analytes, vertical lines indicate a Log_2_ fold-change of +/- 1, and red circles indicate analytes with an fdr corrected p-value ≤ 0.05. Heatmap (bottom panel) representing the fold individual animal fold changes of statistically significant analytes (fdr corrected p-value ≤ 0.05) that also have a group median Log_2_ fold-change ≥ |1|. Individual animal Log_2_ fold-changes were calculated relative to the median values on Study Day 0 of control mice. For each Study Day indicated there are multiple rows, each representing an individual animal while each column represents a different protein analyte. Top panels and bottom panels show H1650 implanted and un-implanted (control) mice, respectively.


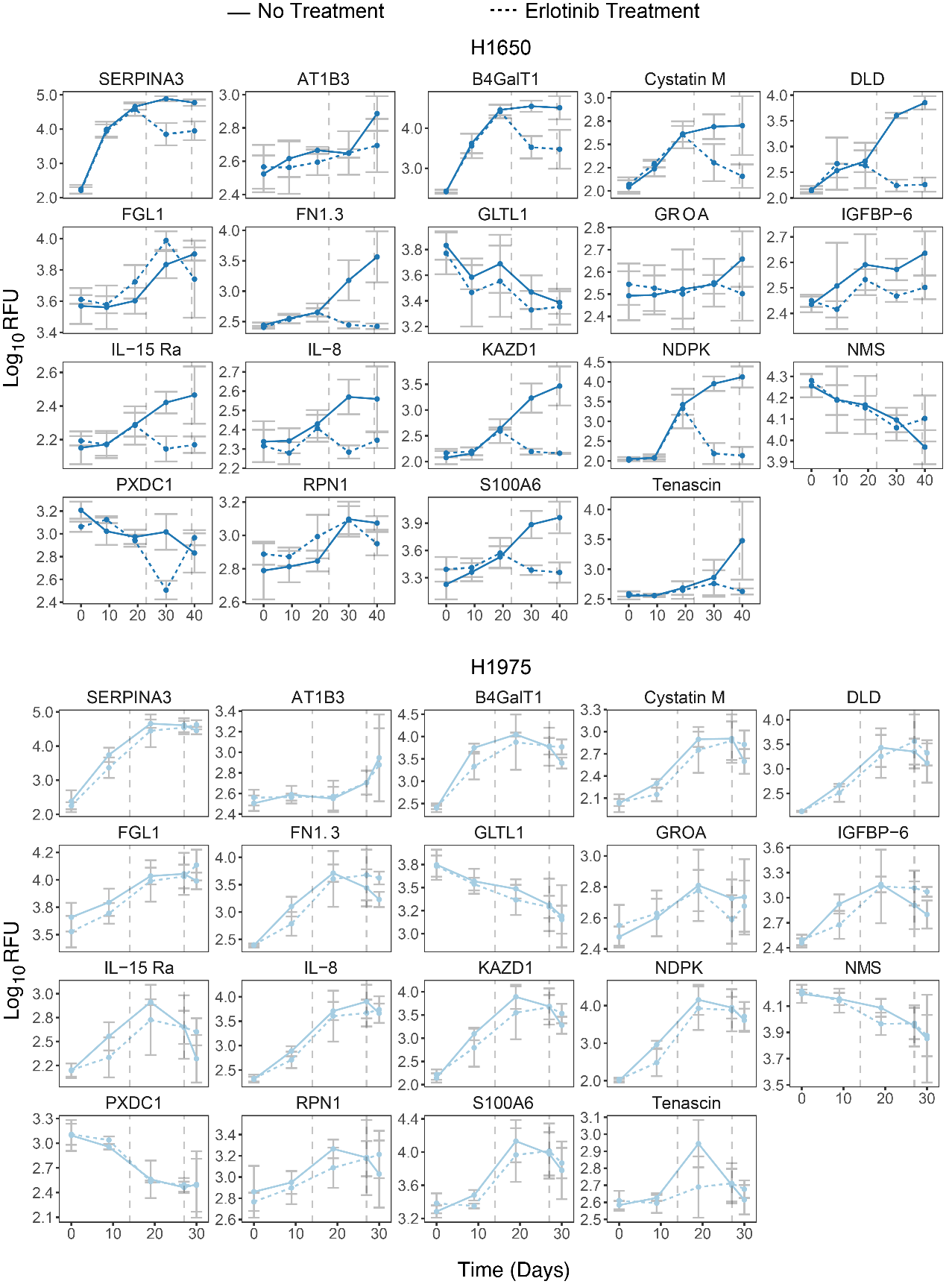


**Figure S2**. Impact of erlotinib treatment on 19-analytes from common NSCLC model. Signal in relative fluorescent units (RFU) versus Study Day for H1650 and H1975 animals with and without treatment with erlotinib. Dashed vertical lines indicate start/stop of erlotinib treatment. Error bars indicate median +/- standard deviation.


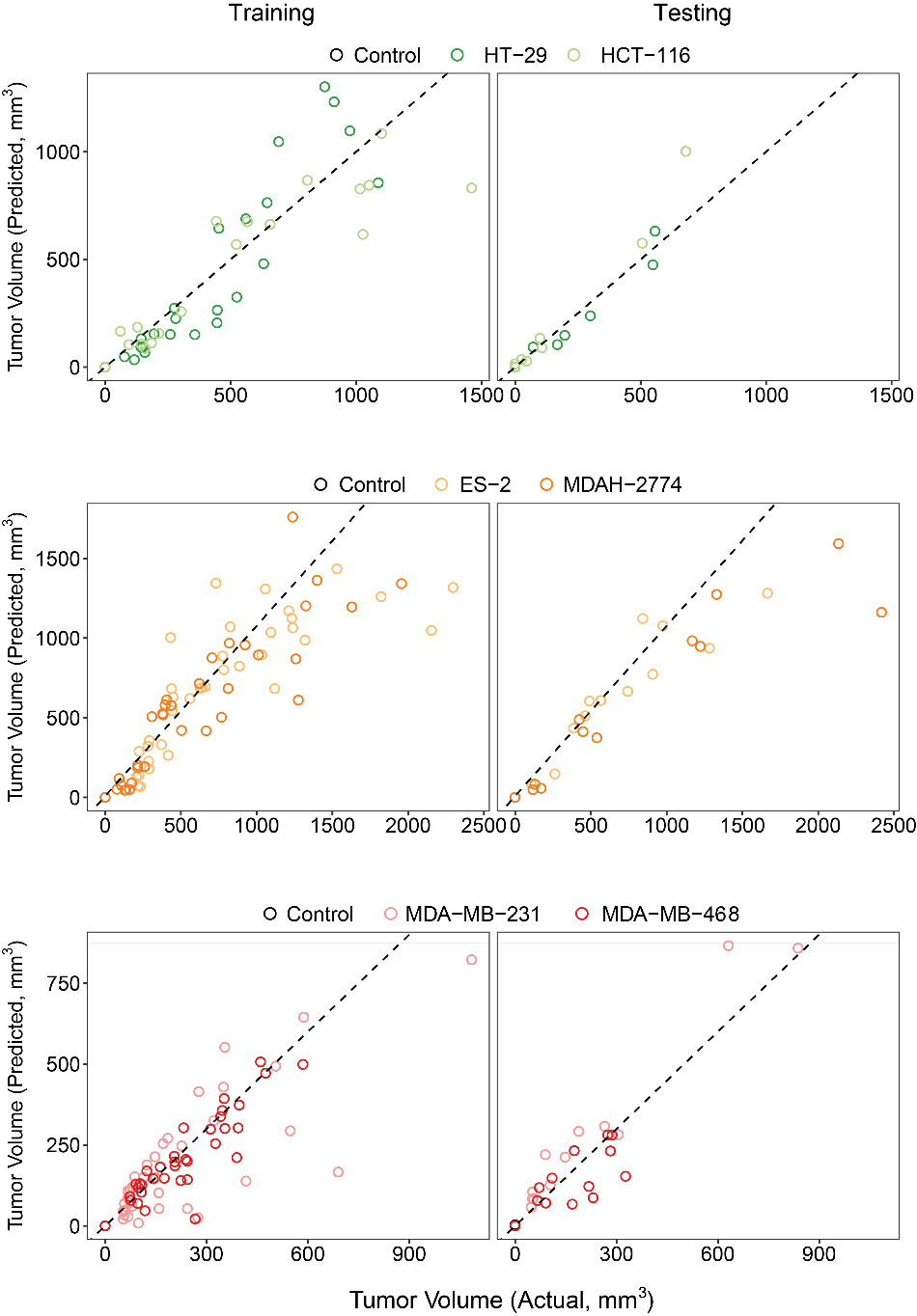


**Figure S3**. Tumor volume prediction in xenograft mice with different tumor types.

Concordance plots (top panels) of actual versus predicted tumor volume trained using 22 common protein markers between colon cancer cell lines HT-29 and HCT-116 for the training (n=6 mice of each) and hold-out test set (n=2 mice of each). Dashed line indicates identity. Same analysis was used for data shown in other panels. Concordance plots (middle panels) of actual versus predicted tumor volume trained using 17 common protein markers between ovarian cancer cell lines ES-2 and MDAH-2774 (n=9 and n=3 for training and testing sets, respectively). Concordance plots (bottom panels) of actual versus predicted tumor volume trained using 24 common protein markers between breast cancer cell lines MDA-MB-231 and MDA-MB-468 (n=6 and n=2 for training and testing sets, respectively).


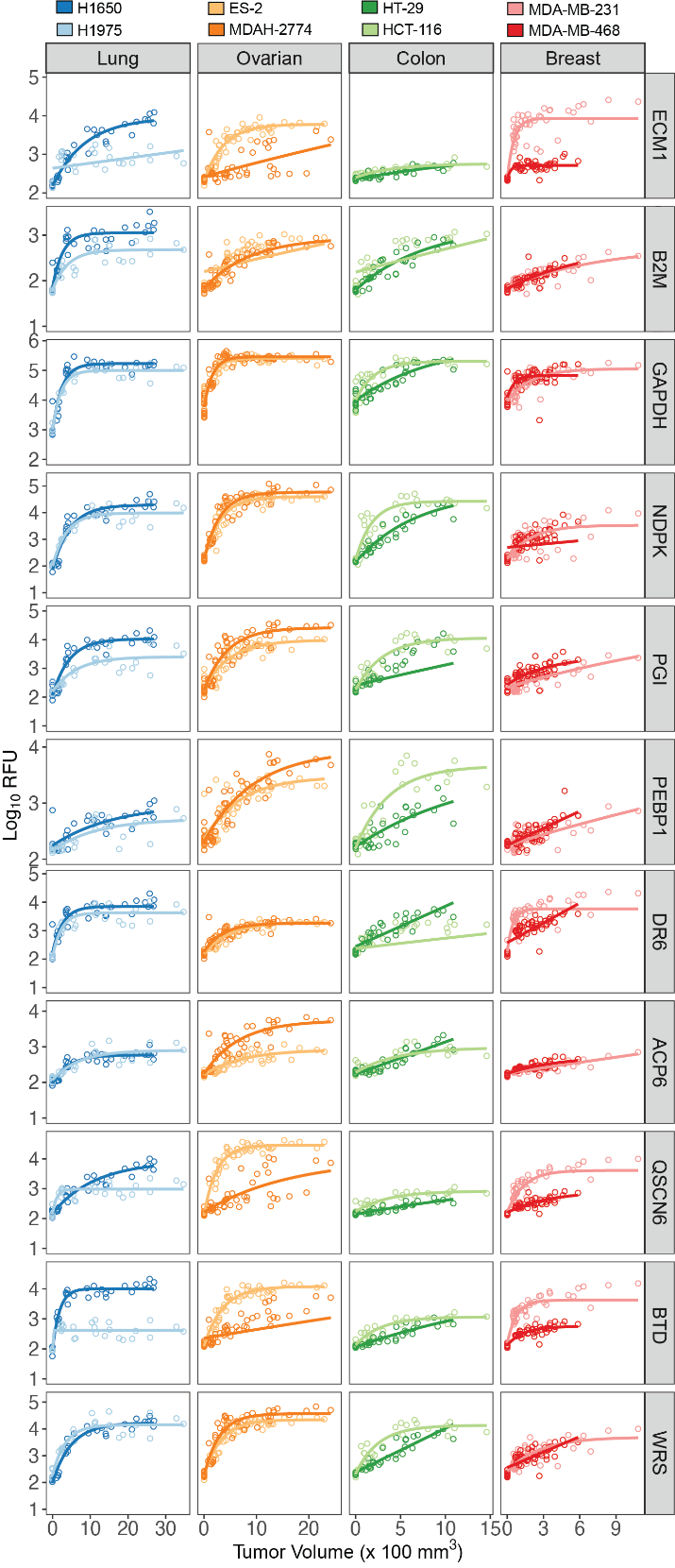


**Figure S4**. Plasma signal versus tumor volume for a set of the 15 common markers across tested cell lines. Signal in relative fluorescent units (RFU) versus tumor volume for a set of 11 common protein markers. The plots for the other 4 biomarkers are shown in **Fig. 4c**. Circles indicate individual SomaScan assay measurements and curves obtained using LOESS regression and smoothing.


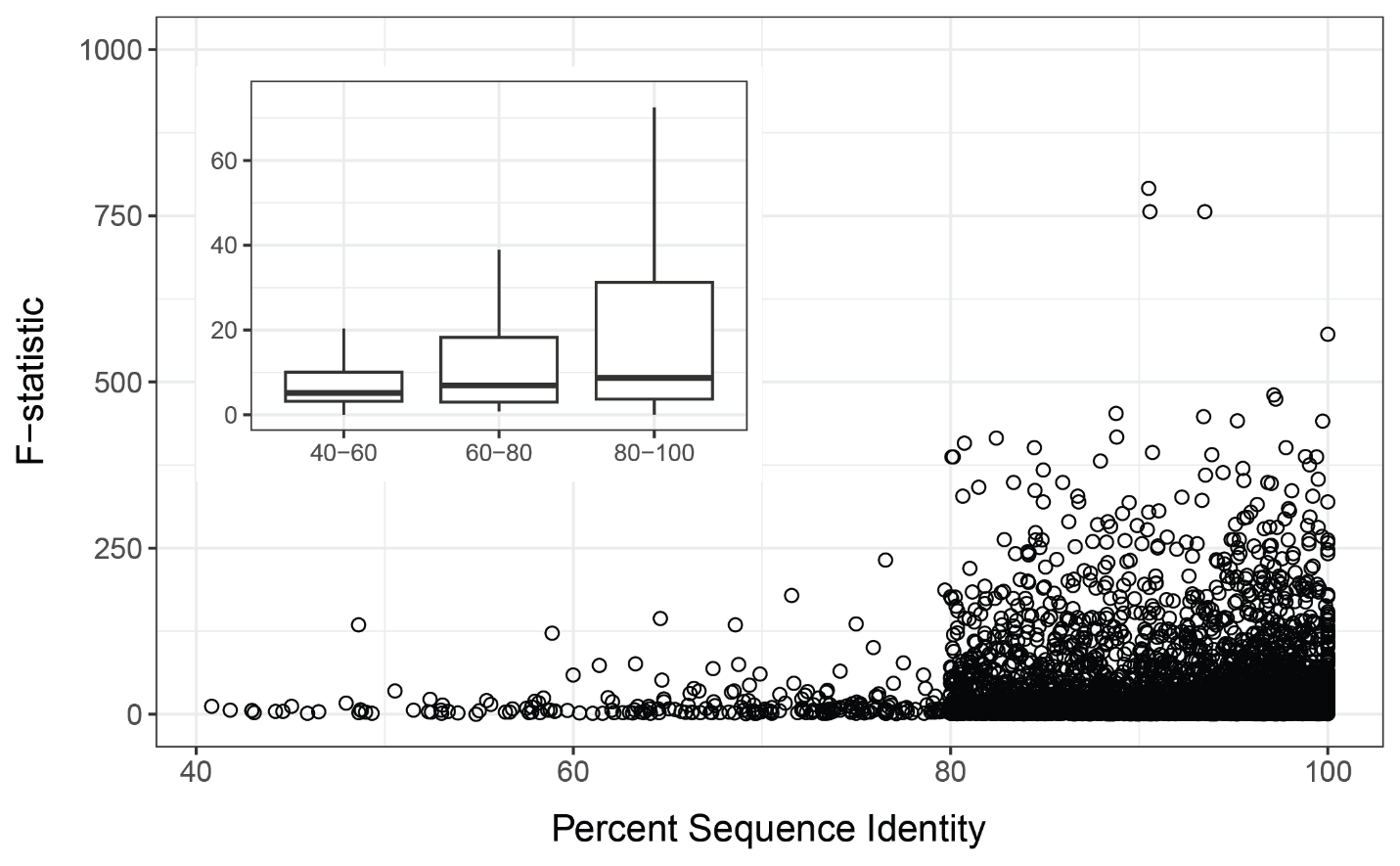


**Figure S5**. Amino acid percent identity versus F-statistic for 4,467 mouse-proteins where the human orthologs are included in the SomaScan assay menu. Inset shows a box and whiskers plot of the same data after grouping by percent identity. Whiskers represent the 10^th^ and 90^th^ percentiles.

**SUPPLEMENTARY NOTE**

**Cross reactivity between mouse and human orthologs in the SomaScan assay**

As the SOMAmer reagents used in the SomaScan assay were selected to recognize human proteins the percentage of the menu that can cross react with mouse proteins is an open question. Mouse and human protein orthologs overall share 78.5% amino acid identity^S58^ supporting the notion that some degree of cross reactivity would be expected. To obtain an initial estimate for the percent cross reactivity within the current SomaScan assay content, we performed an F-test comparing assay variability to the murine population variability. Plasma (EDTA) was obtained from 104 individual mice composed of seventeen male and 17 female mice from each of three different strains (BALB/c, C57BL/6, CD-1) and from one male and one female Swiss Webster mouse. Assay variability was assessed from 104 replicates of an equal volume pool derived from all animals while the population variability was assessed by the results obtained from the 104 individual plasma samples. As with human samples, the SomaScan assay was done in a 3-dilution format (20%, 0.5% and 0.005%).

The population F-statistic, s^2^_pop_ /s^2^_assay_ was calculated for each SOMAmer reagent on the v4.1 SomaScan assay menu. F-statistic values greater than one indicate there is more biological variability in a set of individual samples than in the technical replicates. The F-statistic follows an F-distribution with numerator degrees of freedom of n_pop_ -1 and denominator degrees of freedom of n_rep_ -1 (after dropping flagged samples). The null hypothesis, s^2^_pop_ = s^2^_assay_ can be rejected for values of F-statistic > 1.38 with 95% confidence, and with multiple corrections (n=7,596) > 2.41 with 95% confidence.

The median assay CV across the entire menu was 5.35% with 10^th^ and 90^th^ percentiles of 3.74% and 10.6%, respectively. F-Test results indicated that 83% of the v4.1 SomaScan assay menu had significantly greater population variability than assay variability. Post-test analyses indicate that F-statistic values were not unduly biased by any particular strain of mouse or by sex (data not shown).

Although the F-statistic calculation gives some insight into what percentage of the menu appears to be cross reacting in various species, it does leave some question to how confident we are with the F-statistic in relation to cross-reactivity. To address this question, we obtained the percent sequence identity between mouse and human for 4467 proteins that are included in the SomaScan assay menu. This group had a high sequence identity between the two species (90% average and 92% median). Figure S5 shows the percent sequence identity relative to F-statistic for mice showing a direct relationship between F-statistic and percent sequence identity.

It is important to note that this analysis was performed on a later version of the assay containing a larger menu and a different dilution schema of 20%, 0.5%, 0.005% rather than 5%, 2%, 0.05% used in our study. Although we anticipate that the fraction of analytes that show significant signal above assay noise to be relatively consistent between assay versions, it nonetheless is a limitation of this analysis for this study.

**SupplementaRy References**

S1 Emilsson, V. *et al.* Co-regulatory networks of human serum proteins link genetics to disease. *Science* **361**, 769-773, doi:10.1126/science.aaq1327 (2018).

S2 Emilsson, V. *et al.* Coding and regulatory variants are associated with serum protein levels and disease. *Nat Commun* **13**, 481, doi:10.1038/s41467-022-28081-6 (2022).

S3 Ferkingstad, E. *et al.* Large-scale integration of the plasma proteome with genetics and disease. *Nat Genet* **53**, 1712-1721, doi:10.1038/s41588-021-00978-w (2021).

S4 Pietzner, M. *et al.* Mapping the proteo-genomic convergence of human diseases. *Science* **374**, eabj1541, doi:10.1126/science.abj1541 (2021).

S5 Zhang, J. *et al.* Plasma proteome analyses in individuals of European and African ancestry identify cis-pQTLs and models for proteome-wide association studies. *Nat Genet* **54**, 593-602, doi:10.1038/s41588-022-01051-w (2022).

S6 Sun, B. B. *et al.* Genomic atlas of the human plasma proteome. *Nature* **558**, 73-79, doi:10.1038/s41586-018-0175-2 (2018).

S7 Gordin, D. *et al.* Characterization of Glycolytic Enzymes and Pyruvate Kinase M2 in Type 1 and 2 Diabetic Nephropathy. *Diabetes Care* **42**, 1263-1273, doi:10.2337/dc18-2585 (2019).

S8 Katz, D. H. *et al.* Proteomic profiling platforms head to head: Leveraging genetics and clinical traits to compare aptamer- and antibody-based methods. *Sci Adv* **8**, eabm5164, doi:10.1126/sciadv.abm5164 (2022).

S9 Carayol, J. *et al.* Protein quantitative trait locus study in obesity during weight-loss identifies a leptin regulator. *Nat Commun* **8**, 2084, doi:10.1038/s41467-017-02182-z (2017).

S10 Di Narzo, A. F. *et al.* High-Throughput Characterization of Blood Serum Proteomics of IBD Patients with Respect to Aging and Genetic Factors. *PLoS Genet* **13**, e1006565, doi:10.1371/journal.pgen.1006565 (2017).

S11 Gudjonsson, A. *et al.* A genome-wide association study of serum proteins reveals shared loci with common diseases. *Nat Commun* **13**, 480, doi:10.1038/s41467-021-27850-z (2022).

S12 Sasayama, D. *et al.* Genome-wide quantitative trait loci mapping of the human cerebrospinal fluid proteome. *Hum Mol Genet* **26**, 44-51, doi:10.1093/hmg/ddw366 (2017).

S13 Suhre, K. *et al.* Connecting genetic risk to disease end points through the human blood plasma proteome. *Nat Commun* **8**, 14357, doi:10.1038/ncomms14357 (2017).

S14 Yao, C. *et al.* Genome-wide mapping of plasma protein QTLs identifies putatively causal genes and pathways for cardiovascular disease. *Nat Commun* **9**, 3268, doi:10.1038/s41467-018-05512-x (2018).

S15 Sullivan, K. D. *et al.* Trisomy 21 causes changes in the circulating proteome indicative of chronic autoinflammation. *Sci Rep* **7**, 14818, doi:10.1038/s41598-017-13858-3 (2017).

S16 Tin, A. *et al.* Reproducibility and Variability of Protein Analytes Measured Using a Multiplexed Modified Aptamer Assay. *J Appl Lab Med* **4**, 30-39, doi:10.1373/jalm.2018.027086 (2019).

S17 Zahedi-Amiri, A., Sequiera, G. L., Dhingra, S. & Coombs, K. M. Influenza a virus-triggered autophagy decreases the pluripotency of human-induced pluripotent stem cells. *Cell Death Dis* **10**, 337, doi:10.1038/s41419-019-1567-4 (2019).

S18 Surapaneni, A. *et al.* Identification of 969 protein quantitative trait loci in an African American population with kidney disease attributed to hypertension. *Kidney Int* **102**, 1167-1177, doi:10.1016/j.kint.2022.07.005 (2022).

S19 Stanley, S. *et al.* Comprehensive aptamer-based screening identifies a spectrum of urinary biomarkers of lupus nephritis across ethnicities. *Nat Commun* **11**, 2197, doi:10.1038/s41467-020-15986-3 (2020).

S20 DeBoer, E. M. *et al.* Novel Application of Aptamer Proteomic Analysis in Cystic Fibrosis Bronchoalveolar Lavage Fluid. *Proteomics Clin Appl* **13**, e1800085, doi:10.1002/prca.201800085 (2019).

S21 Huang, G. *et al.* Circulating Biomarkers of Testosterone's Anabolic Effects on Fat-Free Mass. *J Clin Endocrinol Metab* **104**, 3768-3778, doi:10.1210/jc.2019-00505 (2019).

S22 Christensson, A. *et al.* The Impact of the Glomerular Filtration Rate on the Human Plasma Proteome. *Proteomics Clin Appl* **12**, e1700067, doi:10.1002/prca.201700067 (2018).

S23 Sher, A. A., Glover, K. K. M. & Coombs, K. M. Zika Virus Infection Disrupts Astrocytic Proteins Involved in Synapse Control and Axon Guidance. *Front Microbiol* **10**, 596, doi:10.3389/fmicb.2019.00596 (2019).

S24 Coombs, K. M., Simon, P. F., McLeish, N. J., Zahedi-Amiri, A. & Kobasa, D. Aptamer Profiling of A549 Cells Infected with Low-Pathogenicity and High-Pathogenicity Influenza Viruses. *Viruses* **11**, doi:10.3390/v11111028 (2019).

S25 Raffield, L. M. *et al.* Comparison of Proteomic Assessment Methods in Multiple Cohort Studies. *Proteomics* **20**, e1900278, doi:10.1002/pmic.201900278 (2020).

S26 Cuvelliez, M. *et al.* Circulating proteomic signature of early death in heart failure patients with reduced ejection fraction. *Sci Rep* **9**, 19202, doi:10.1038/s41598-019-55727-1 (2019).

S27 Jalal, D. *et al.* Endothelial Microparticles and Systemic Complement Activation in Patients With Chronic Kidney Disease. *J Am Heart Assoc* **7**, doi:10.1161/jaha.117.007818 (2018).

S28 Webber, J. *et al.* Proteomics analysis of cancer exosomes using a novel modified aptamer-based array (SOMAscan™) platform. *Mol Cell Proteomics* **13**, 1050-1064, doi:10.1074/mcp.M113.032136 (2014).

S29 Fong, T. G. *et al.* Identification of Plasma Proteome Signatures Associated With Surgery Using SOMAscan. *Ann Surg* **273**, 732-742, doi:10.1097/sla.0000000000003283 (2021).

S30 Graumann, J. *et al.* Multi-platform Affinity Proteomics Identify Proteins Linked to Metastasis and Immune Suppression in Ovarian Cancer Plasma. *Front Oncol* **9**, 1150, doi:10.3389/fonc.2019.01150 (2019).

S31 Mehan, M. R. *et al.* Protein signature of lung cancer tissues. *PLoS One* **7**, e35157, doi:10.1371/journal.pone.0035157 (2012).

S32 Rhodes, C. J. *et al.* Plasma proteome analysis in patients with pulmonary arterial hypertension: an observational cohort study. *Lancet Respir Med* **5**, 717-726, doi:10.1016/s2213-2600(17)30161-3 (2017).

S33 Lertudomphonwanit, C. *et al.* Large-scale proteomics identifies MMP-7 as a sentinel of epithelial injury and of biliary atresia. *Sci Transl Med* **9**, doi:10.1126/scitranslmed.aan8462 (2017).

S34 Tanaka, T. *et al.* Plasma proteomic signature of age in healthy humans. *Aging Cell* **17**, e12799, doi:10.1111/acel.12799 (2018).

S35 Lazarev, V. F., Guzhova, I. V. & Margulis, B. A. Glyceraldehyde-3-phosphate Dehydrogenase is a Multifaceted Therapeutic Target. *Pharmaceutics* **12**, doi:10.3390/pharmaceutics12050416 (2020).

S36 Zhang, J. Y. *et al.* Critical protein GAPDH and its regulatory mechanisms in cancer cells. *Cancer Biol Med* **12**, 10-22, doi:10.7497/j.issn.2095-3941.2014.0019 (2015).

S37 Babady, N. E., Pang, Y. P., Elpeleg, O. & Isaya, G. Cryptic proteolytic activity of dihydrolipoamide dehydrogenase. *Proc Natl Acad Sci U S A* **104**, 6158-6163, doi:10.1073/pnas.0610618104 (2007).

S38 Fermo, E. *et al.* Clinical and Molecular Spectrum of Glucose-6-Phosphate Isomerase Deficiency. Report of 12 New Cases. *Front Physiol* **10**, 467, doi:10.3389/fphys.2019.00467 (2019).

S39 Erez, A. *et al.* Requirement of argininosuccinate lyase for systemic nitric oxide production. *Nat Med* **17**, 1619-1626, doi:10.1038/nm.2544 (2011).

S40 Huberts, D. H. & van der Klei, I. J. Moonlighting proteins: an intriguing mode of multitasking. *Biochim Biophys Acta* **1803**, 520-525, doi:10.1016/j.bbamcr.2010.01.022 (2010).

S41 Adam, I. *et al.* Upregulation of tryptophanyl-tRNA synthethase adapts human cancer cells to nutritional stress caused by tryptophan degradation. *Oncoimmunology* **7**, e1486353, doi:10.1080/2162402x.2018.1486353 (2018).

S42 Jin, M. Unique roles of tryptophanyl-tRNA synthetase in immune control and its therapeutic implications. *Exp Mol Med* **51**, 1-10, doi:10.1038/s12276-018-0196-9 (2019).

S43 Jobin, P. G. *et al.* Matrix metalloproteinases inactivate the proinflammatory functions of secreted moonlighting tryptophanyl-tRNA synthetase. *J Biol Chem* **294**, 12866-12879, doi:10.1074/jbc.RA119.009584 (2019).

S44 Pataskar, A. *et al.* Tryptophan depletion results in tryptophan-to-phenylalanine substitutants. *Nature* **603**, 721-727, doi:10.1038/s41586-022-04499-2 (2022).

S45 Attwood, P. V. & Muimo, R. The actions of NME1/NDPK-A and NME2/NDPK-B as protein kinases. *Lab Invest* **98**, 283-290, doi:10.1038/labinvest.2017.125 (2018).

S46 Puts, G. S., Leonard, M. K., Pamidimukkala, N. V., Snyder, D. E. & Kaetzel, D. M. Nuclear functions of NME proteins. *Lab Invest* **98**, 211-218, doi:10.1038/labinvest.2017.109 (2018).

S47 Lake, D. F. & Faigel, D. O. The emerging role of QSOX1 in cancer. *Antioxid Redox Signal* **21**, 485-496, doi:10.1089/ars.2013.5572 (2014).

S48 Qasba, P. K., Ramakrishnan, B. & Boeggeman, E. Structure and function of beta -1,4-galactosyltransferase. *Curr Drug Targets* **9**, 292-309, doi:10.2174/138945008783954943 (2008).

S49 Li, J. *et al.* Crystal structures and biochemical studies of human lysophosphatidic acid phosphatase type 6. *Protein Cell* **4**, 548-561, doi:10.1007/s13238-013-3031-z (2013).

S50 Zempleni, J., Hassan, Y. I. & Wijeratne, S. S. K. Biotin and biotinidase deficiency. *Expert Review of Endocrinology & Metabolism* **3**, 715-724, doi:10.1586/17446651.3.6.715 (2008).

S51 Rajkumar, K. *et al.* Understanding perspectives of signalling mechanisms regulating PEBP1 function. *Cell Biochem Funct* **34**, 394-403, doi:10.1002/cbf.3198 (2016).

S52 Lee, K. M. *et al.* Extracellular matrix protein 1 regulates cell proliferation and trastuzumab resistance through activation of epidermal growth factor signaling. *Breast Cancer Res* **16**, 479, doi:10.1186/s13058-014-0479-6 (2014).

S53 Yin, H. *et al.* Extracellular matrix protein-1 secretory isoform promotes ovarian cancer through increasing alternative mRNA splicing and stemness. *Nat Commun* **12**, 4230, doi:10.1038/s41467-021-24315-1 (2021).

S54 Ren, X., Lin, Z. & Yuan, W. A Structural and Functional Perspective of Death Receptor 6. *Front Pharmacol* **13**, 836614, doi:10.3389/fphar.2022.836614 (2022).

S55 Prizment, A. E. *et al.* Circulating Beta-2 Microglobulin and Risk of Cancer: The Atherosclerosis Risk in Communities Study (ARIC). *Cancer Epidemiol Biomarkers Prev* **25**, 657-664, doi:10.1158/1055-9965.Epi-15-0849 (2016).

S56 Lee, H. N., Jeong, M. S. & Jang, S. B. Molecular Characteristics of Amyloid Precursor Protein (APP) and Its Effects in Cancer. *Int J Mol Sci* **22**, doi:10.3390/ijms22094999 (2021).

S57 Pandey, P. *et al.* Amyloid precursor protein and amyloid precursor-like protein 2 in cancer. *Oncotarget* **7**, 19430-19444, doi:10.18632/oncotarget.7103 (2016).

S58 Waterston, R. H. *et al.* Initial sequencing and comparative analysis of the mouse genome. *Nature* **420**, 520-562, doi:10.1038/nature01262 (2002).
